# Effect of Temperature Ageing on Injection Molded High-Density Polyethylene Parts Modified by Accelerated Electrons

**DOI:** 10.3390/ma15030742

**Published:** 2022-01-19

**Authors:** Ales Mizera, Miroslav Manas, Pavel Stoklasek

**Affiliations:** Faculty of Applied Informatics, Tomas Bata University in Zlin, Nad Stranemi 4511, 760 05 Zlin, Czech Republic; manas@utb.cz (M.M.); pstoklasek@utb.cz (P.S.)

**Keywords:** high-density polyethylene, temperature stability/ageing, radiation cross-linking, injection molding technology, mechanical properties

## Abstract

The temperature ageing of high-density polyethylene (HDPE) modified by accelerated electrons was studied. Commodity plastic HDPE was used as a basic polymer material which was modified by radiation cross-linking. This polymer was used because of its excellent processability and chemical resistance. Plastic injection molding technology was used for the production of test specimens. These specimens were modified with the dose of radiation 33, 66, 99, 132, 165, and 198 kGy. The prepared specimens were tested to determine: gel content, degree of swelling, temperature stability, and changes in mechanical properties after temperature ageing. The results were determined by scanning electron microscopy (SEM) analysis on the fracture surfaces. The results of this study confirm that modification of HDPE by radiation cross-linking has a significant effect on increasing temperature stability. It has been shown that HDPE modified by radiation cross-linking can withstand temperatures exceeding the melting point of the original HDPE for a short-term.

## 1. Introduction

Polymers are possibly used in all scientific and technical fields, but also in common industries concerning each of us. We encounter them daily and take them as a part of our daily lives. Commodity polymers, e.g., polyethylene (PE), have a very important place in the packaging industry. PE is widely used for its low cost, easy processability, and high chemical resistance. It is used for its irreplaceable properties in pipes and packaging, but also as technical parts. A typical representative of commodity polymers is high-density polyethylene (HDPE), which excels with its good wear resistance, high fracture toughness, chemical stability, corrosion resistance, light weight, etc. [1,2,3,4]. The biggest disadvantage of HDPE is its relatively low temperature stability, which makes it impossible to apply this material where there may be a risk of the temperature rising above the tolerable limit. Exceeding this limit would lead to the collapse of this system, which is unacceptable. Another disadvantage is the relatively low stiffness of HDPE itself, which can be modified with fillers such as glass fibers [5,6,7,8,9,10].

If injection molding technology is used on HDPE in technical applications, it is necessary to modify HDPE in order to improve especially its temperature stability, or to achieve further improvement of the required properties—e.g., improvement of surface properties, etc. There are a number of modifications that can improve the properties of HDPE, but the greatest effect on increasing the temperature stability has been shown by radiation cross-linking. The advantage of this technique is that HDPE mostly cross-links even without the addition of a cross-linking agent. Thus, the modification can take place independently of the production technology of the HDPE part itself. It is not necessary to change the already established technology, there will be only additional modification by accelerated electrons, which will cause cross-linking of the HDPE part and modification of certain properties [11,12,13,14,15,16,17,18,19,20].

Many scientific papers deal with the study of the thermal and mechanical properties of modified PE. A frequently used modification technique is the addition of nanoparticles, carbon fibers, etc. In particular, the effect on the tribological properties and microstructure of a given composite is determined. Pelto et al. [21] studied the influence of different types of nanofillers on the tribological properties of the surface of HDPE composites. In some cases, the coefficient of friction and wear has been reduced with the addition of a small amount of nanofiller [21,22,23,24,25,26,27]. In certain cases, it is appropriate to modify the thermal conductivity, which is generally poor for all polymeric materials, as these are thermal insulators. There are many properties that can be modified. On the one hand, the addition of various substances improves the desired property, but on the other hand, it can also impair other properties, which are also important. It is always necessary to find a certain optimum [28,29,30,31,32,33].

Whatever type of HDPE modification was used and the final product matches the application, there is another important parameter, and that is the processability and cost of the final product. Related to this there is the competitiveness of the modification and its use in mass production. As mentioned at the beginning, there are certain modifications (beta, gamma radiation) that can be applied without problems only after production, such as injection molding technology. However, for other modifications it is necessary to consider that the addition of fillers, for example, leads to certain problems with the dosing and homogenization of the filler, which can result in different properties of the injected products. Therefore, it is necessary to take into account what modification will be used when planning the production technology. Only in this way can we achieve HDPE injection-molded products can be used in technical applications as components with high temperature stability [34,35,36,37,38].

Many studies deal with UV or thermal ageing of pure or variously modified PE. They determine thermal, mechanical, and other properties that change depending on the mechanism of degradation. Whatever the type of ageing, the effect of thermal ageing on the mechanical properties of injected HDPE parts modified by radiation cross-linking (accelerated electrons) has not yet been published [39,40,41]. Xu et al. studied the thermal ageing of silane cross-linked PE stabilized with varying amounts of distearyl thiodipropionate, using FTIR spectroscopy and DSC techniques [42]. Weingrill et al. [43] worked on long-term stabilization and ageing of HDPE with various stabilization systems. In this research, thermo-oxidative stability was studied. The degradation behavior of HDPE was monitored by DSC, FTIR, and polarized light microscopy [43].

The presented article deals with the study of temperature stability of injection molded HDPE parts, which were subsequently modified by radiation cross-linking (accelerated electrons). Based on the determination of temperature stability of modified HDPE, the mechanical properties after temperature ageing were studied. The results were determined by scanning electron microscopy (SEM), which showed minimal surface disturbance after temperature loading. This article provides a new perspective on the study of temperature stability and temperature ageing of polymers other than by conventional thermal methods (DSC, FTIR, TG, etc.).

## 2. Materials and Methods

### 2.1. Material and Specimen Preparation

Radiation cross-linked high-density polyethylene (HDPE) modified by accelerated electrons was used to investigate the effect of temperature ageing on mechanical behavior. For a basic HDPE we used a commercially known product with the trade name HDPE 25055 E, which was supplied by DOW (Midland, MI, USA). This polymer belongs to the polyolefin group, which is easy to process, has excellent chemical resistance, but unfortunately it has a relatively low temperature stability.

**Figure 1:**
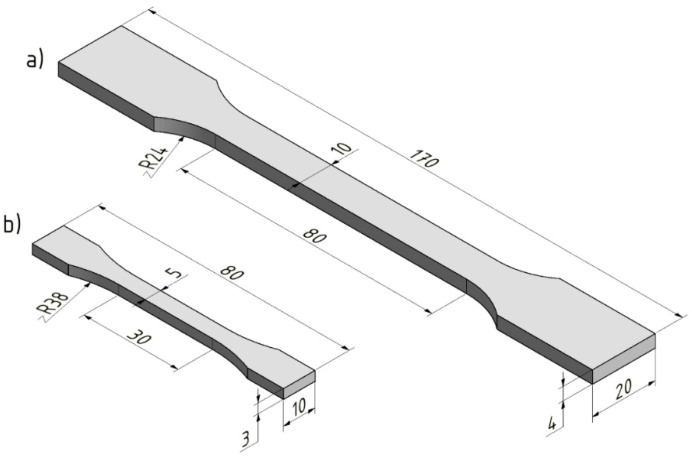
Shape and dimensions of tested specimens: (**a**) tensile test, (**b**) tensile impact test.

The test samples were produced by two injection molding machines: Arburg Allrounder 420 C 1000-350 with screw diameter 40 mm (Loßburg, Germany); and Arburg Allrounder 170 U with screw diameter 15 mm (Loßburg, Germany). Process parameters, which can be seen in Table 1, were set according to the recommendation of the material’s manufacturer. The shape and dimensions of the specimens that were tested (Figure 1), were governed by the CSN EN ISO 527-1 and CSN EN ISO 8256 standard.

### 2.2. Radiation Cross-Linking by Accelerated Electrons

One of the possibilities to improve temperature stability is the method of radiation cross-linking. In this research, the accelerated electrons were used as a source of radiation energy. This process was done under standard atmospheric conditions and room temperature in cooperation with BGS Beta-Gamma-Service, located in Germany. The source of electrons is a hot-cathode made from wolfram, then these electrons are accelerated in a strong electric field and a high vacuum of a Rhodotron high-voltage accelerator, which presented the maximum energy of 10 MeV (Tongeren, Belgium). The range of the dosages was set in compliance with experience gained from industrial practice to 33, 66, 99, 132, 165, and 198 kGy. Each accelerator cycle exposed the test sample to the radiation dose of 33 kGy. The adequate radiation dose was determined by a Nylon FTN 60-00 dosimeter (Far West Technology, Inc., Goleta, CA, USA). The analysis of absorbed radiation dose by the dosimeter was performed with a Genesys 5 spectrophotometer (Thermo Fisher Scientific, Waltham, MA, USA), in accordance with the ASTM 51261 standard.

### 2.3. Gel Content Test and Degree of Swelling

A gel (content) test is performed in order to determine the non-dissolved gel content of the given material—according to the ASTM D 2765 standard—Test Method C. A portion of 0.5 g (irradiated HDPE) weighed with a precision of five decimal places on a “SWISS MADE EP 125 SM” weighing apparatus (Dietikon, Switzerland) was mixed with 100 mL of solvent. Xylene was used in this case because it dissolves the amorphous part of this material, and the cross-linked part does not dissolve. The mixture was extracted for 24 h. Then, the solutes were separated by distillation. After removing the residual xylene, the cross-linked extract was dried for 8 h, in a vacuum, at 100 °C. The dried and cooled residue was weighed again with a precision of five decimal places and compared to the original weight of the portion. The result is stated in percentage as the degree of cross-linking

(1) $$G_{i}=\frac{m_{3}-m_{1}}{m_{2}-m_{1}}100$$

where *G_i_* is the degree of cross-linking of each specimen expressed in percentage; *m*_1_ is the weight of the cage and lid in milligrams; *m*_2_ is the total of the weights of the original specimen, cage, and lid in milligrams; and *m*_3_ is the total of the weights of the residue specimen, cage, and lid in milligrams.

The swelling test was performed on the basis of the CSN ISO 1817 standard, which is primarily intended for elastomers. The swelling test was performed in xylene at 120 °C for HDPE. The initial weight of the test specimens was about 0.2 g. The weight of the test specimens was recorded in time at 1, 2, 4, and 7 h intervals when steady state had occurred. Five specimens were used for each measurement; arithmetic mean and standard deviation were used for gel content test and degree of swelling.

### 2.4. Temperature Stability

The temperature stability test consisted of two different tests of observation of dimensional change and deformation at temperature. In the first test, the test specimens were placed over their entire surface on the non-stick surface of the steel plate. The temperature chamber and the steel plate itself were preheated to 220 °C. After stabilizing the temperature, the test specimens were placed on a steel plate and placed in a temperature chamber for 15 min at 220 °C. After this time, the steel plate with the deformed samples was pulled out of the temperature chamber and images were taken. Subsequently, there was a gradual cooling at room temperature. In the second test, visual observation was carried out in a temperature chamber where pictures were taken at 30 min intervals. This technique is not as precise or as sensitive as a thermo-mechanical analysis but, from the macroscopic point of view, deformation under temperature influences is visible at first sight. The test specimens were loaded in the horizontal position, with a bending moment from their own weight and the bending moment from the force acting on the body-end (1.5 N·mm). The collapse due to its own weight in the vertical position was also studied. Both temperature stability tests were repeated 3 times to verify the obtained results.

### 2.5. Vicat Softening Temperature

The Vicat softening temperature was measured on an HDT 6 Vicat CEAST type 6921 instrument (CEAST, Turin, Italy). The test was performed in accordance with CSN EN ISO 306/A50. The test specimens loaded with a force of 10 N were immersed in oil, which had been heated at a rate of 50 °C/h from ambient temperature since the beginning of the test. The recording of the Vicat softening temperature took place automatically when the tip with a cross-section of 1 mm^2^ penetrated to 1 mm of the test specimen. Five specimens were used for each measurement; arithmetic mean and standard deviation were used for Vicat softening temperature determination.

### 2.6. Thermo-Mechanical Analysis

Temperature stability was assessed using a thermo-mechanical analysis in the penetration mode. The thermo-mechanical properties were measured using a Perkin-ElmerDMA 7e thermal analyzer, Waltham, MA, USA, which was used for the thermo-mechanical analysis, heated from 50 to 250 °C, at 20 °C/min, and held for 1 min at 50 °C. This precise temperature stability evaluation of the polymers (e.g., irradiated/cross-linked polymers) records the displacement of the probe with a diameter 1 mm at a loading of 160 mN, which penetrates into the heated material (cut sample dimensions 10 × 10 × 4 mm^3^), in a set range of temperatures. The resulting curves of thermo-mechanical analysis are the average values from 5 measurements.

### 2.7. Tempereature Ageing

**Figure 2:**
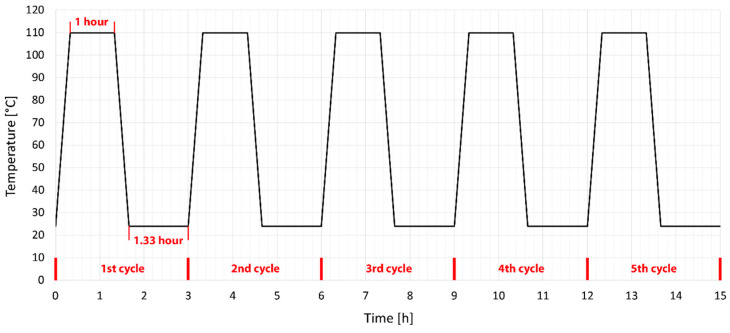
Setting temperature ageing cycle of HDPE under the melting point.

**Figure 3:**
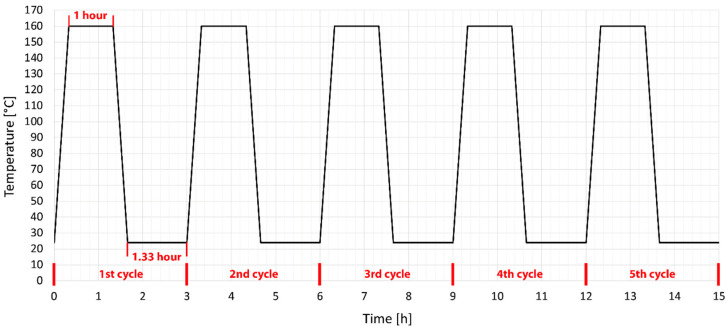
Setting temperature ageing cycle of HDPE above the melting point.

Unmodified and HDPE test specimens modified by radiation cross-linking were subsequently subjected to temperature ageing in a temperature chamber. The temperature was chosen to be in the range of melting point of unmodified HDPE. A temperature 25 °C lower and higher than the melting point of unmodified HDPE (135 °C) was chosen. The Figure 2 and Figure 3 graphically show the temperature cycles by which the individual test specimens were temperature loaded. The test specimens were tempered according to predefined temperature cycles between two 6 mm thick steel plates with a non-stick surface. This method of heating was chosen to avoid possible deformations due to different temperatures on the bottom and top of the specimens. At the end of the cycle, the test specimens were sorted and stored for further testing of mechanical properties.

### 2.8. Tensile Test

A ZWICK 1456 tensile machine (Ulm, Germany) was used for the estimation of tensile behavior. Measurements were carried out according to the ISO 527 standard at ambient (23 °C) temperature with crosshead speed of 50 mm/min. Fifteen samples were tested and their E-modulus, ultimate tensile strength, and strain at break values were evaluated in TestExpertII and MS Excel software. Conditioning was taken for five days at a temperature of 23 °C and relative humidity of 50%. Arithmetic mean and standard deviation are used in all figures.

### 2.9. Tensile Impact Test

The tensile impact test was carried out on Zwick HIT50P equipment (Zwick, Ulm, Germany) at ambient temperature of 23 °C according to standard ISO 8256. 50 J impact hammer and 120 g crosshead were used on this test. Fifteen specimens (Figure 1) were tested, and their ultimate impact tensile strength values were evaluated in TestExpert II and MS Excel programs. Arithmetic mean and standard deviation were used as the statistical parameters in this measurement.

### 2.10. Scanning Electron Microscopy (SEM)

The structure of the fracture surfaces was examined using a JEOL 7500F scanning electron microscope (JEOL, Tokyo, Japan). Fracture surfaces for SEM were prepared by breaking the test specimens after cooling in liquid nitrogen. The test specimens with fracture surfaces were glued to the targets with a dispersion glue and plated with gold in an argon atmosphere on a Balzers sputtering device (Oerlikon Balzers, Balzers, Lichtenstein). SEM images were scanned in *.bmp format with the PC SEM program. The accelerating voltage used was 15 kV and the working distance (WD) was from 6 to 9 mm.

## 3. Results

Plastic injection molding technology is a very precise and progressive method of manufacturing of plastic parts. This technology is especially suitable for large series, but in some cases, it is also used for piece production. It all depends on the acquisition costs for the tool/mold and the use of the final product. The resulting properties of the final product will depend both on the process conditions of production and mainly on the material used. Materials with excellent processing conditions usually have lower temperature stability and vice versa. HDPE with excellent processing conditions was used for our study, however, the resistance to temperature ageing is limiting. One of the possibilities to improve the temperature stability of HDPE is the modification by radiation cross-linking (accelerated electrons). With this modification, the basic property—namely, thermoplasticity—is lost, but from the commodity polymer, there is obtained the material with the properties of engineering polymers. This is shown in the following results of temperature stability and selected mechanical properties, such as tensile test and impact tensile test.

### 3.1. Gel Content Test and Degree of Swelling

The gel content test is one of the ways to assess at least seemingly the density of the network after radiation cross-linking. In the soluble phase—the sol is extracted in xylene and after drying only the cross-linked component ‘gel’ remains. With this test, we can determine the percentage of the cross-linked component, but the result says nothing about the density and character of the network. We can find this method in many scientific articles because it is relatively easy to perform in laboratory conditions, but the explanatory power of this test is debatable, which is based on the principle of this test. To describe the behavior of the modified polymers in more detail, a xylene swelling test was used, which proved to be more suitable for determining the network density. The results show more obvious differences between the modified polymers than in the previous method.

#### 3.1.1. Gel Content Test

**Figure 4:**
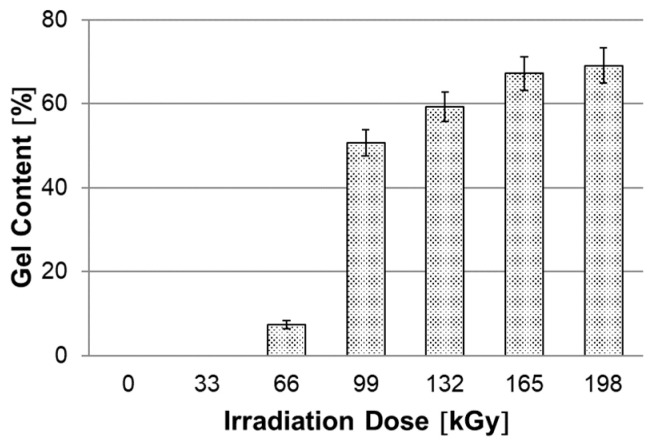
Dependence of gel content on irradiation dose.

The gel content at each dose of irradiation is shown in Figure 4. Unmodified HDPE was completely dissolved in xylene. In HDPE modified with the lowest dose used, 33 kGy, the value of the gel was unable to be measured. The reason is low cross-linking and the formation of so-called microgels, which were smaller than the mesh of the cage in which the sample was inserted. At a radiation dose of 66 kGy, the minimum value of the gel (7.4%) was measured. However, a more significant increase occurred only at a radiation dose of 99 kGy. Furthermore, the gel content increased only slightly with the radiation dose, up to a maximum value of 69.1% at an irradiation dose of 198 kGy. This test shows that with increasing irradiation doses, up to a maximum of 198 kGy used, the gel content (proportion of cross-linked component) increases slightly. However, with this test, we are not able to characterize the density of the network. Therefore, the swelling test of modified HDPE in xylene was used as a supplementary test (described below).

#### 3.1.2. Degree of Swelling

**Figure 5:**
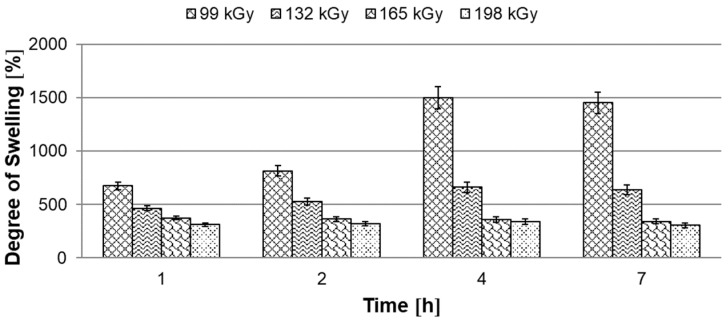
Dependence of swelling degree on time.

Based on the evaluation of the gel content, the degree of swelling of the modified HDPE was measured with a radiation dose higher than 99 kGy as can be seen in Figure 5. After 7 h in xylene, full saturation and maximum swelling occurred. HDPE modified with a radiation dose of 99 kGy swelled by 1500%. Already with another dose of 132 kGy radiation, a rapid decrease in swelling was recorded, by more than a half. A similar swelling value (300%) was measured at irradiation doses of 165 and 198 kGy. Measurements of gel content and degree of swelling indicate that the highest two radiation doses used are the optimal choice for HDPE modification.

### 3.2. Temperature Stability

The temperature stability of the polymer is usually lower than that of metallic or ceramic materials. The main advantage of the polymers is the relatively good processability with low energy requirements. HDPE thermoplastic material was used in this study. Without any modification of this material, with increasing temperature, gradual softening (loss of mechanical properties) occurs until the complete melting of the material at the melting point (HDPE 135 °C). There are several modification options that can increase the temperature stability of thermoplastics. One possibility is radiation cross-linking by beta (electron) radiation. The benefits of this modification have been known for many years, but not all questions have been answered. One of them is the multiple temperature loading of HDPE modified by beta (electron) radiation with subsequent evaluation of the changes of mechanical properties. In this chapter, the influence of the amount of absorbed beta (electron) radiation on the short-term temperature stability of HDPE is studied.

#### 3.2.1. Short-Term Temperature Load at 220 °C

**Figure 6:**
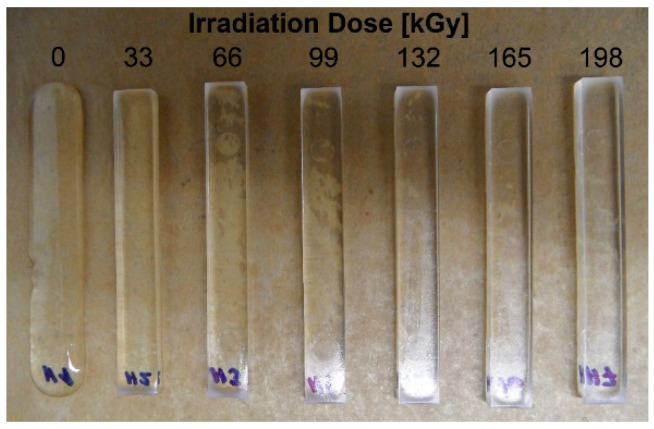
Short-term temperature stability at 220 °C.

A short-term test (15 min) at 220 °C was used as the first temperature stability test of the modified HDPE beta (electron) radiation. Figure 6 shows the condition after this exposure. As can be seen, the unmodified HDPE has completely melted even in this short period of time. At low radiation doses (33 and 66 kGy), only the ends of the test specimens were melted/rounded, the shape of the specimens was otherwise preserved. No shape changes were observed at higher doses (99–198 kGy).

#### 3.2.2. Visual Observation in the Temperature Chamber

**Figure 7:**
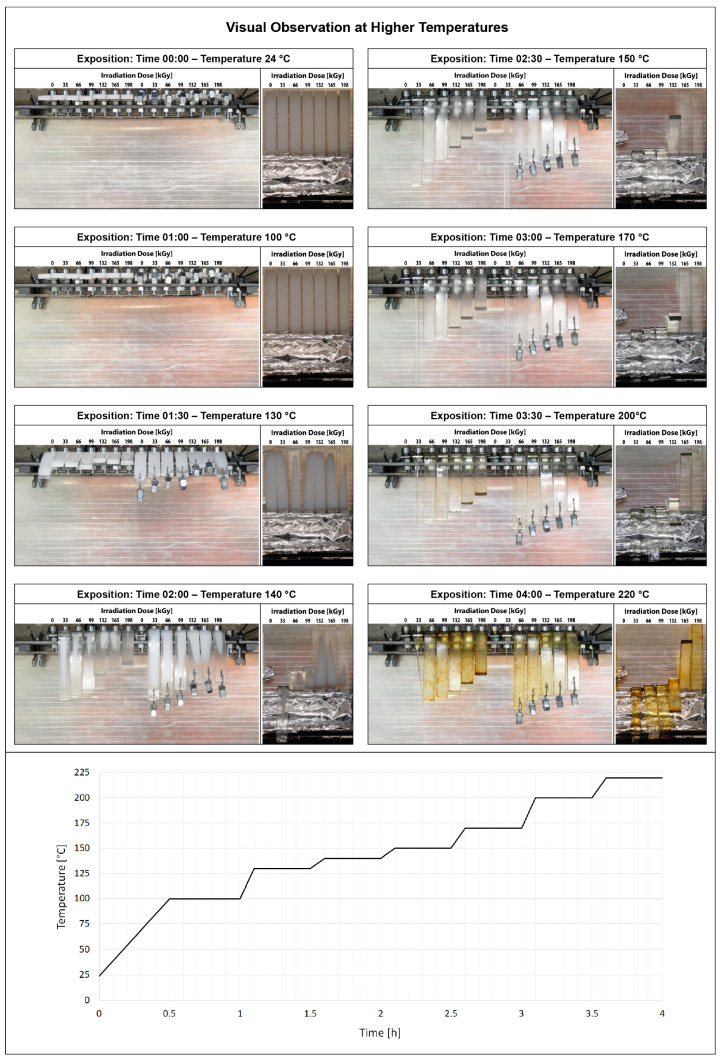
Visual observation of temperature stability in temperature chamber.

The second temperature stability test was the observation of test specimens in a temperature chamber according to a predefined temperature profile, which is shown in the Figure 7 below. Unmodified and test specimens modified by radiation cross-linking were placed in a temperature chamber in three configurations: horizontal, horizontal with load, and vertical (this condition was observed at the start and at 1 h). As the temperature increases, the test specimens gradually soften. This condition is evident at 1 h 30 min at 130 °C when the chamber temperature begins to approach the melting point of unmodified HDPE. The test specimens begin to deform under their weight. The test specimens still remain in a vertical position without collapsing. After exceeding the melting point of 135 °C, the horizontal test specimens of unmodified HDPE melted. Other horizontal specimens deform more and more under their weight, but still retain their shape and dimensions. Vertical test specimens with low radiation doses of 33, 66, and 99 kGy collapsed; however, samples with higher radiation doses (132–198 kGy) are still in a vertical position. At a temperature of 150 °C, the 33 kGy modified specimen displayed creep. Next, the specimen modified by radiation dose of 132 kGy begins to collapse. Test specimens modified with a radiation dose of 165 and 198 kGy still withstood this temperature. With increasing time and temperature, at a temperature of 200 °C, there is a slight deviation from the vertical position of the test body modified with a radiation dose of 165 kGy. Furthermore, it can be observed that all modified test specimens no longer undergo further deformation, but there is gradual thermo-oxidation, which is manifested by a change in the color of the specimens (yellow). This phenomenon is clearly visible after 4 h at a temperature of 220 °C, when the modified test specimens are already yellow-brown in color with surface defects. The result of this test is that highly irradiated HDPE test specimens with a radiation dose of 198 kGy deformed under load, but did not collapse in the vertical test.

### 3.3. Vicat Softening Temperature

**Figure 8:**
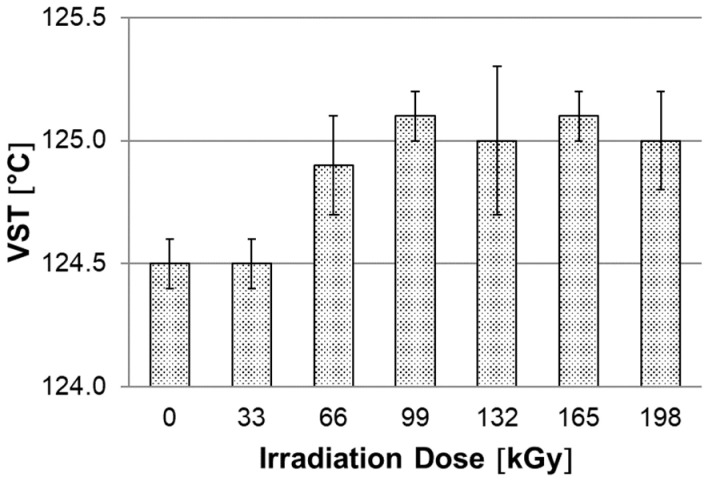
Dependence of Vicat softening temperature (VST) on irradiation dose.

Another test of temperature stability is the Vicat softening temperature (VST), which is very often used in practice for its simplicity and speed of determining the Vicat softening temperature. In the Figure 8, only small differences can be observed between unmodified and HDPE modified by accelerated electrons test specimens. Unmodified HDPE and HDPE modified with a radiation dose of 33 kGy reached a similar VST of 124.5 °C. In contrast, all other test specimens modified with a radiation dose from 66 to 198 kGy reached about 0.5 °C higher VST values. According to the standard, a load of 10 N was used on individual test specimens. This load is relatively high, which is reflected in the sensitivity of this test. On the other hand, it can be seen that under higher loads, both unmodified and modified HDPE test specimens lose their temperature stability at a similar temperature.

### 3.4. Thermo-Mechanical Analysis

**Figure 9:**
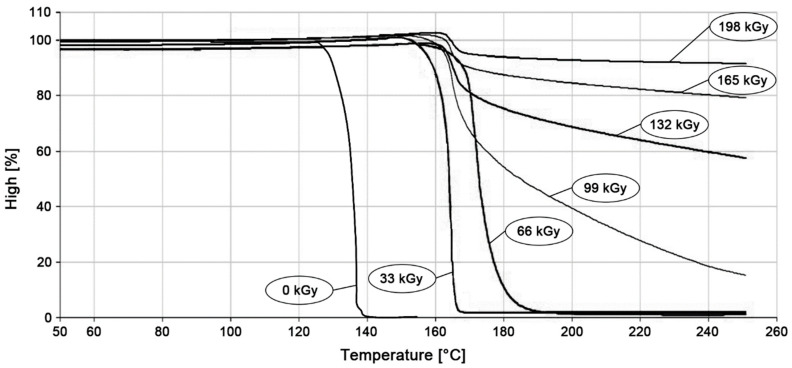
Graphic record of thermo-mechanical analysis.

Thermo-mechanical analysis (TMA) was used as the last temperature stability test, which is very sensitive to any change in network density after HDPE radiation cross-linking. This is mainly due to the relatively low load of 0.16 N that acts on the tip and the very sensitive sensors that can accurately record the position of the tip in the sample. As can be seen from the Figure 9, the tip penetrated through the unmodified HDPE through the melting point of this material. In a test specimen modified with a radiation dose of 33 kGy, the tip fully penetrated at a temperature of 165 °C. Furthermore, the tip penetrated the HDPE modified with a radiation dose of 66 kGy at a temperature of 180 °C. The other modified HDPE specimens did not have complete penetration, but only partial penetration, even at a temperature of 250 °C. With increasing radiation doses, the penetration of the tip decreased up to the maximum used radiation dose of 198 kGy (10% at a temperature of 250 °C). The results of this test correspond to the results of measuring the gel content and the degree of swelling, as well as to the visual observation test in the temperature chamber.

### 3.5. Tensile Test

Temperature stability tests have shown that HDPE modified by radiation cross-linking, especially with high radiation doses around 198 kGy, can withstand temperatures of up to 250 °C for short periods. At this temperature, strong thermo-oxidation already takes place, which causes degradation. This chapter describes the effect of temperature loading (ageing) on mechanical properties, more precisely on the tensile E-modulus, ultimate tensile strength and strain at break. These properties were measured for both unloaded and temperature loaded polymers after one and five cycles at 110 °C and 160 °C (Figure 2 and Figure 3).

**Figure 10:**
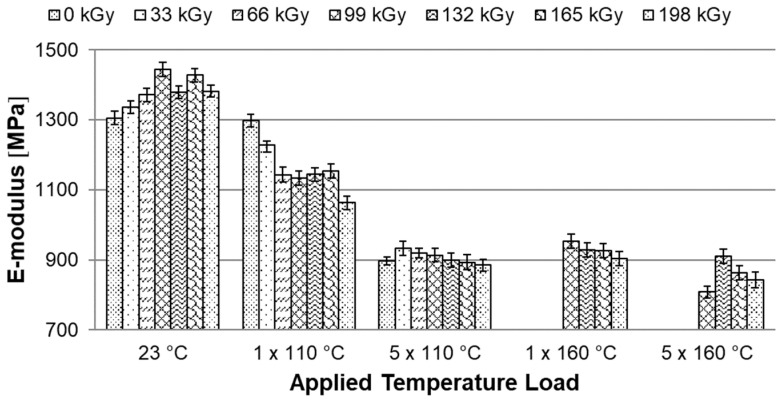
Dependence of E-modulus on applied temperature load.

The Figure 10 shows the dependence of the E-modulus on the applied temperature load. In temperature unloaded test specimens, the E-modulus increases slightly with increasing radiation up to a radiation dose of 99 kGy, then with a further increasing radiation dose, there is a slight decrease and stabilization at 1382 ± 17 MPa at the maximum used radiation dose of 198 kGy. Compared to unmodified HDPE, this is an increase of 6% in the E-modulus. Already after the first cycle of temperature loading at 110 °C, the E-modulus decreases in all modified HDPE specimens. The E-modulus did not change for unmodified HDPE after the first cycle at 110 °C. HDPE modified with a radiation dose of 198 kGy showed a rapid decrease in E-modulus, both after five cycles at 110 °C and after one and five cycles at 160 °C. Here, the E-modulus of all tested specimens decreased by about 31%.

**Figure 11:**
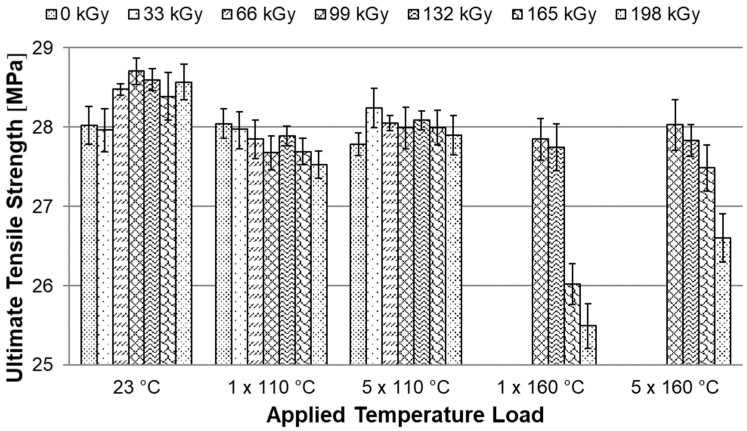
Dependence of ultimate tensile strength on applied temperature load.

Another monitored parameter was ultimate tensile strength. Here in Figure 11, it can be observed that the HDPE modification by radiation cross-linking has a positive effect. The ultimate tensile strength increased by about 2%. Due to the temperature load at 110 °C, there was no significant decrease in the ultimate tensile strength. However, after a temperature load of 1 × 160 °C, the specimens with the radiation doses of 165 and 198 kGy demonstrate a decrease in ultimate tensile strength by 9% compared to the temperature unloaded unmodified HDPE. After the fifth cycle at 160 °C, these test specimens also decreased. However, this value is relatively low and therefore we can state that the applied temperature load does not have a significant effect on the ultimate tensile strength of modified HDPE specimens.

**Figure 12:**
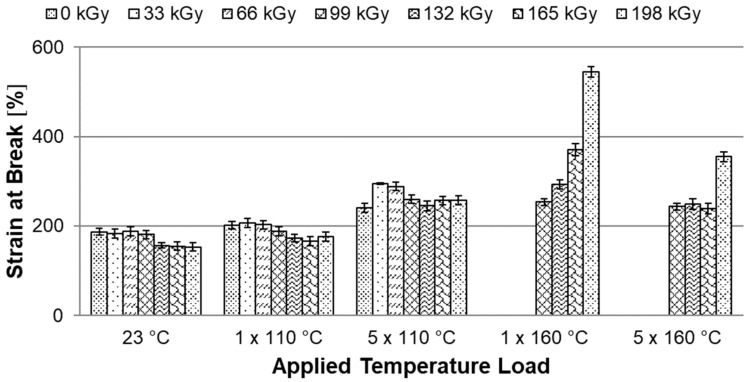
Dependence of the strain at break on applied temperature load.

The last parameter monitored was strain at break. The Figure 12 shows that none of the tested specimens has a significantly lower strain at break after temperature loading than unmodified HDPE without temperature loading. On the contrary, at a temperature load of 1 × 160 °C, the strain at break of the test specimen with a radiation dose of 198 kGy increased by 191%.

### 3.6. Tensile Impact Test

**Figure 13:**
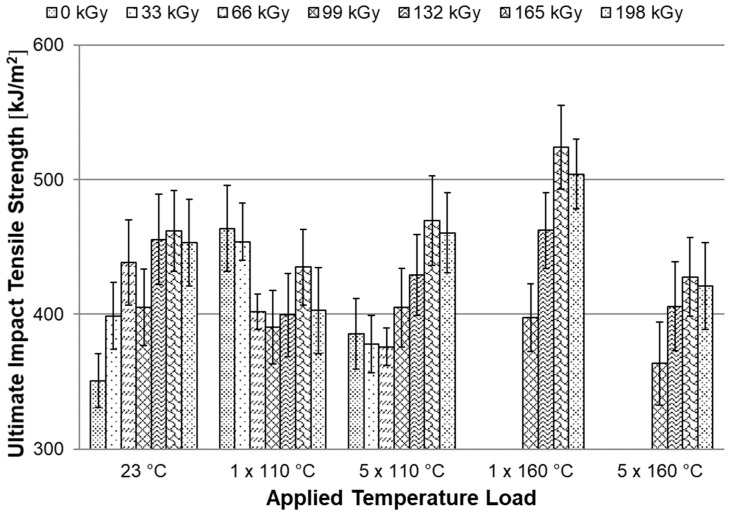
Dependence of the ultimate impact tensile strength on applied temperature load.

The last test in this study was the tensile impact test. In Figure 13, it can be seen that the ultimate impact tensile strength increases slightly with the radiation dose. Furthermore, it can be seen from the Figure 13 that there was no deterioration in impact properties of temperature loaded test specimens. This shows that modification of HDPE by radiation cross-linking is one of the suitable modification options to increase the temperature stability of injection-molded HDPE products.

### 3.7. Scanning Electron Microscopy (SEM)

**Figure 14:**
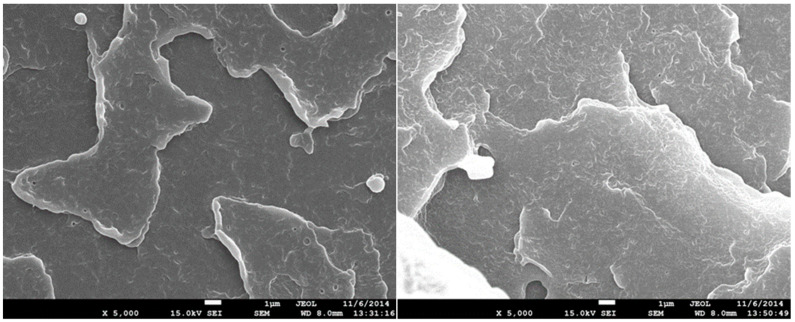
SEM 5000×–fracture surface of unmodified (**left**) and HDPE modified with radiation dose 198 kGy (**right**).

**Figure 15:**
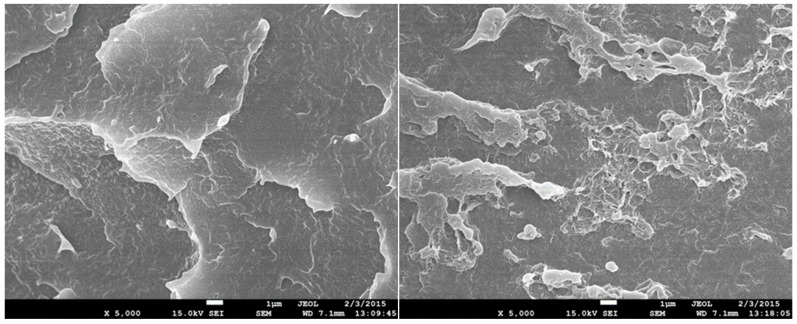
SEM 5000×–fracture surface of unmodified (**left**) and HDPE modified with radiation dose 198 kGy (**right**) after temperature loading at 110 °C.

**Figure 16:**
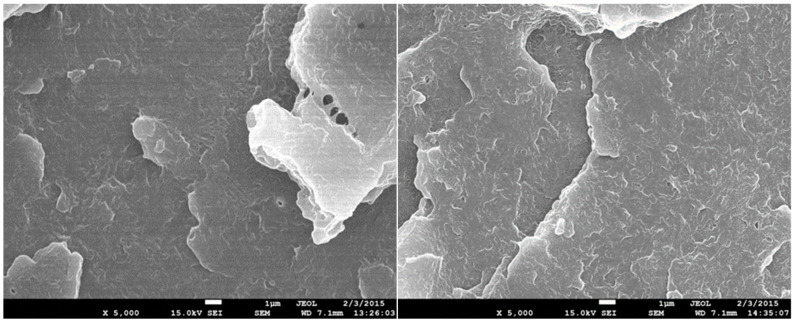
SEM 5000×–fracture surface of unmodified (**left**) and HDPE modified with radiation dose of 198 kGy (**right**) after five cycles of temperature loading at 110 °C.

**Figure 17:**
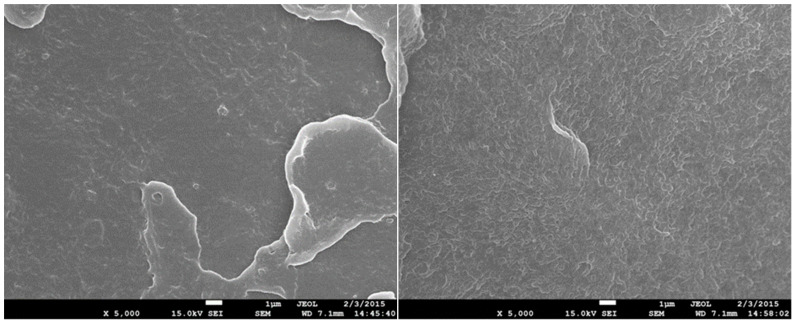
SEM 5000×–fracture surface of HDPE modified with radiation dose of 198 kGy after temperature loading at 160 °C (**left**) and after five cycles of temperature loading at 160 °C (**right**).

The structure of test specimens unmodified and modified by radiation cross-linking with a radiation dose of 198 kGy before and after temperature loading was investigated by SEM on fracture surfaces that were gilded in an argon atmosphere. Images were recorded at 5000× magnification for all test specimens measured. In Figure 14, the structure of temperature unloaded test specimens can be seen. The structure of the unmodified test specimen is shown on the left and the structure of the HDPE modified with a radiation dose of 198 kGy is on the right. A certain change in the structure of the fracture surface can be observed. A larger number of microscopic cracks can be observed in the HDPE modified with a radiation dose of 198 kGy, which can be caused by the interruption of the cross-linked chains. In Figure 15 and Figure 16, it is possible to see the structure after one and five cycles of temperature loading at 110 °C. It is possible to observe more microscopic cracks than in the specimens without temperature loading. However, at a temperature load of 160 °C, there is apparently a smoother fracture surface with nanoscopic cracks (Figure 17). From this analysis, it is possible to say that the changes of the structure fracture surface are relatively small, which complements the results of measurement mechanical properties after temperature loading without apparent changes.

## 4. Discussion

HDPE is one of the commodity plastics that are widely used especially in the packaging industry due to its excellent properties. In particular, it excels in chemical resistance to alcohols, salt solutions, alkalis and acids, acetone, isopropyl alcohol, and in the short-term to gasoline. It also excels in superb processability, it can be processed with practically all plastics technologies. Unfortunately, it also has its disadvantages, and these are relatively low temperature stability and poor adhesiveness, due to its non-polar character. However, these negative properties of HDPE can be improved by certain types of modification. One of these modifications is radiation cross-linking. For this purpose, accelerated electrons were used in the present study. The advantage of this modification is that it only takes place on the final product, e.g., on HDPE parts that do not require a cross-linking agent. This modification can also take place on other materials that do not allow cross-linking without a cross-linking agent, which must already be present in the granulate to be processed, where subsequently, modification by radiation cross-linking is possible—for example, PP or PA.

HDPE modified by radiation cross-linking can be classified as a construction material in terms of certain properties, which is very important for injection-molded parts that are used in technical fields. The use of such a relatively cheap material with this added value seems evidently preferable.

In this study, it was found that the gel content of the modified HDPE increases with increasing radiation dose, a significant increase occurred only at a dose of 99 kGy, then the gel content increased slightly. The gel content of 69.1% was measured at the highest radiation dose of 198 kGy. The measurement of the degree of swelling showed a similar trend when the degree of swelling decreased with increasing radiation dose. In the next part, we dealt with the temperature stability of HDPE modified by radiation cross-linking. Two observation methods were used, the first method was short-term heating of test specimens to 220 °C, the second was the observation of deformation of test specimens according to the set temperature profile in the temperature chamber. Both tests confirmed that radiation cross-linking has a significant effect on increasing temperature stability. Short-term modified HDPE which was irradiated (from a radiation dose of 99 kGy) and could withstand temperatures higher than 200 °C without dimensional changes. A slight increase in VST (0.5 °C) was also demonstrated for the test specimens modified with a radiation dose from 66 kGy to 198 kGy. The biggest differences in temperature stability were shown in the thermo-mechanical analysis, which is very sensitive to any changes in the density of the network modified by HDPE radiation cross-linking. Here, the results correlate with the visual observation test in the temperature chamber, where the temperature stability increases with increasing dose. Modified HDPE with the highest radiation dose of 198 kGy proved to be the most resistant in terms of temperature stability.

It has been found that HDPE test specimens modified by radiation cross-linking with a radiation dose higher than 99 kGy can withstand short-term temperature loads higher than the melting point of unmodified HDPE. It is now very important to find out whether there are rapid changes in the mechanical properties after temperature loading, which would limit the use of this material in technical applications. Therefore, the modified HDPE test specimens were subjected to temperature ageing at 110 °C and 160 °C. The mechanical properties were measured on temperature unloaded test specimens and after one and five cycles at each temperature (see temperature profile in the Figure 2 and Figure 3). A tensile test was performed and based on statistical evaluation, the following parameters were monitored: E-modulus, ultimate tensile strength, and strain at break. The temperature load had the greatest effect on the E-modulus. Hereafter, with a temperature load at 110 °C and 160 °C, there was a decrease of 31% in the E-modulus compared to the unmodified temperature unloaded HDPE. Furthermore, there was a slight decrease in ultimate tensile strength in test specimens modified with a radiation dose of 165 and 198 kGy at a temperature load of 160 °C. The decrease was 9% compared to unmodified temperature-free HDPE. However, this reduction is relatively low and no radical deterioration can be expected after this short-term temperature load, even at 160 °C. In contrast, strain at break increased in all temperature-loaded test specimens by up to 191%, which could cause stress relaxation in the test specimens. The temperature-loaded test specimens were finally subjected to an impact tensile test, where the ultimate impact tensile strength did not deteriorate in any of the temperature load cases. In fact, the properties have suddenly improved. Additionally, no signs of significant damage to the internal structure of HDPE were found on the fracture surfaces tested before and after temperature loading, which was studied by SEM. These results indicate that modification of HDPE by accelerated electrons is one of the options that can increase the temperature stability of HDPE without significantly changing the mechanical properties and without damaging the internal structure.

## 5. Conclusions

HDPE is a very widespread material, especially in the packaging industry, due to its properties. The advantage is easy processability—e.g., by injection molding technology—which can be used to produce precise products in large series. In order for this material to be used in other industrial areas, it must be modified to increase the temperature resistance, which is relatively low for raw HDPE. One possibility is modification by radiation crosslinking, which was used in this study.

Based on the research results, the following conclusions can be drawn:

- Radiation crosslinking using accelerated electrons has a significant effect on the temperature stability of the tested HDPE;
- Results of thermos-mechanical analysis and visual observation of changes during the tests show an increasing temperature stability with increasing radiation dose;
- For a short term, HDPE modified with a radiation dose of 198 kGy can withstand temperatures higher than 200 °C without dimensional/shape changes;
- There was no significant deterioration of the tested mechanical properties after temperature ageing;
- After temperature ageing, the observed impact properties even improved;
- SEM also did not reveal significant changes in the structure of the fracture surface after temperature ageing.

Further research on this issue will focus on the long-term temperature stability of modified HDPE by accelerated electrons.

## Figures and Tables

**Table 1:** Injection molding parameters.

Processing Conditions	420 C	170 U
Injection Rate (mm/s)	60	40
Injection Pressure (MPa)	80	60
Holding Pressure (MPa)	60	40
Holding Time (s)	30	30
Cooling Time (s)	35	30
Mold Temperature (°C)	40	40
**Plastic Unit Temperature Bands**		
Zone 1 (°C)	205	180
Zone 2 (°C)	210	190
Zone 3 (°C)	225	195
Zone 4 (°C)	230	200

## Data Availability

The data presented in this study are available on request from the corresponding author.
